# A New Bevacizumab Carrier for Intravitreal Administration: Focus on Stability

**DOI:** 10.3390/pharmaceutics13040560

**Published:** 2021-04-15

**Authors:** Daniela Chirio, Elena Peira, Simona Sapino, Giulia Chindamo, Simonetta Oliaro-Bosso, Salvatore Adinolfi, Chiara Dianzani, Francesca Baratta, Marina Gallarate

**Affiliations:** Department of Drug Science and Technology, University of Torino, Via P. Giuria 9, 10125 Torino, Italy; daniela.chirio@unito.it (D.C.); elena.peira@unito.it (E.P.); giulia.chindamo@unito.it (G.C.); simona.oliaro@unito.it (S.O.-B.); chiara.dianzani@unito.it (C.D.); francesca.baratta@unito.it (F.B.); marina.gallarate@unito.it (M.G.)

**Keywords:** lipid nanoparticles, microemulsion, bevacizumab, structure stability, biocompatibility, intravitreal injection, drug delivery

## Abstract

Bevacizumab (BVZ) is a monoclonal antibody that binds to human vascular endothelial growth factor A (VEGF-A) and inhibits the interaction between VEGF-A and VEGF receptors, thus blocking the angiogenesis. Repeated intravitreal injections of BVZ for the treatment of ocular pathologies that present an excessive proliferation results in a low patience compliance. BVZ is specially indicated for the treatment of diabetic and degenerative retinopathy. In the present study, we designed lipid nanoparticles (NPs) as a BVZ sustained drug delivery system for reducing the frequency of administration. We used a simple and highly efficient procedure, “Cold dilution of microemulsions”, to obtain spherical NPs with mean diameters of 280–430 nm, Zeta potentials between −17 and −31 mV, and drug entrapment efficiencies between 50 to 90%. This study focused on the biochemical and biophysical stabilities of BVZ after entrapment in NPs. SDS-PAGE electrophoretic analysis and circular dichroism, dynamic light scattering, and scanning electron microscopy were used to characterize BVZ-loaded NPs. The biocompatibility was assessed by in vitro cell compatibility studies using the ARPE-19 cell line. Thus, in this work, a stable BVZ-loaded system was obtained. In addition, several studies have shown that BVZ is released slowly from the lipid matrix and that this system is biocompatible. The results are promising and the developed NPs could be exploited to create a new, potentially effective and minimally invasive treatment of intraocular diseases.

## 1. Introduction

Bevacizumab (AVASTIN^®^, BVZ) is a recombinant humanized monoclonal immunoglobulin antibody, with antihuman vascular endothelial growth factor (VEGF) activity, approved as an antitumoral agent in the treatment of several tumors such as colorectal and lung cancer, and renal cell carcinoma [1]. Its activity is due to the ability to bind the human vascular endothelial growth factor A (VEGF-A) inhibiting its interaction with VEGF receptor tyrosine kinases and blocking the angiogenesis, and the growth of new vessels from pre-existing vasculature, which is a critical step in tumor progression [2].

In addition to tumor angiogenesis, an excessive new blood vessels growth is also characteristic of eye conditions leading to blindness, such as age-related macular degeneration [3], proliferative diabetic retinopathy [4], and macular edema. Anti-VEGF therapy has improved the quality of life for many patients affected by the aforementioned diseases, as the inhibition of intraocular VEGF is the only therapeutic strategy that can preserve vision; pegaptanib, ranibizumab, and aflibercept have been approved for intravitreal administration. On the other hand, BVZ, which has been proven to be effective against the same diseases, is currently used off-label in intravitreal administration for many months or even years, because its suspension or discontinuation may cause the recurrence of neovascularization [5,6]. Indeed, intravitreal injection is an administration route for the therapy of many posterior segment ocular diseases [7]. As the half-life of BVZ is 3–4 days [8], multiple injections are needed, leading to a high risk of complications such as endophthalmitis, intraocular inflammation, rhegmatogenous retinal detachment, and ocular hemorrhage. Another disadvantage is the pain associated with inserting needles into eyes [9]. Therefore, there is a considerable need to develop a drug delivery system able to release BVZ in a slow and long-lasting manner to reduce the frequency of administration and make BVZ intravitreal therapy less invasive and less harmful, consequently enhancing patient compliance.

In the literature, some BVZ-loaded nanosystems suitable for intravitreal injection are described. Some of the numerous literature data are reported hereafter. Abrishami et al. [10] prepared BVZ-loaded liposomes, vesicular systems composed of an aqueous core enclosed by phospholipid bilayers, which were injected with conventional soluble BVZ, in the right and left eyes of albino rabbits, respectively. The intravitreal injection of BVZ encapsulated in liposomes was tolerated well, and the drug clearance in vitreous from liposomal formulations was slower than the soluble form was. Moreover, the developed liposomes provided sufficient concentrations of the therapeutic drug for >6 weeks, suggesting their exploitation as an intravitreal delivery system.

Polymeric micro/nanoparticles (NPs) represent a further strategy to achieve a prolonged intravitreal release of BVZ. Li et al. [11] studied BVZ-loaded nano- and microspheres of poly (D, L-lactide-co-glycolide) and poly (ethylene glycol)-b-poly (D, L-lactic acid), respectively. Although the BVZ activity still needs to be tested in vivo using animal models, the in vitro results indicate that BVZ can be released from these systems in a sustained way for over 90 days and that the release rate could be modified by changing the drug/polymer ratio.

Lu and colleagues [12] prepared BVZ-chitosan NPs with an emulsification evaporation method to study the effects of their intravitreal injection on the pathological morphology of the retina and on the expression of the VEGF protein and VEGF mRNA in the retina of diabetic rats. Chitosan is a natural, nontoxic, biodegradable polysaccharide with attractive physico-chemical and functional versatile properties, allowing its broad applications in chitosan-based micro/NPs. BVZ-chitosan NPs lasted longer than free-BVZ solution, effectively inhibiting both VEGF expression and diabetic retinopathy progression.

Yandrapu et al. [13] used supercritical infusion and pressure quench technology to prepare BVZ-coated PLA NPs encapsulated into porosifying PLGA microparticles. The in vitro release of BVZ from this system was sustained for up to 4 months and the released drug maintained its monomeric form, conformation, and activity.

Despite the encouraging results obtained regarding the sustained release of BVZ, the main disadvantages of the aforementioned experiments are related to either the use of nonbiodegradable matrices or the application of complex production technologies requiring sophisticated equipment.

Keeping this in mind, we aimed to develop BVZ-loaded NPs—employing highly biocompatible substances permitted for parenteral use—intended for intravitreal administration and to prepare them using a simple and reproducible process named the “cold microemulsion dilution” technique.

The starting point is the preparation of an O/W microemulsion (µE) using a partially water-miscible solvent as an oil phase; following µE water dilution, the solubilization of the organic solvent in water occurs, with the consequent precipitation of NPs [14,15]. Such a technique, unlike most methods described in the literature, does not require high temperatures or sonication or pH variations that might negatively influence drug stability or entrapment. It combines the advantages of the emulsion solvent diffusion technique with the high stability and the super-solvent properties of microemulsive systems.

In the formulation step, particular attention was given to verify the maintenance of the BVZ structural integrity after entrapment. Indeed, BVZ might undergo alterations during the formulation steps, as a result of the interaction with some components of the μE.

The obtained NPs were characterized for their physico-chemical characteristics, for the BVZ in vitro release rate and, finally, for their in vitro biocompatibility on human retinal pigment epithelial ARPE-19 cells by cell viability assay.

## 2. Materials and Methods

### 2.1. Materials

Propylene glycol was purchased from ACEF (Fiorenzuola d′Arda, Italy); benzyl alcohol, Sepharose^®^CL 4B, ethyl acetate, sodium sulfate, sodium dodecyl sulfate, phosphate buffer saline (PBS) tablets, trichloroacetic acid, M199 medium, heparin, MTT, VEGF-α, crystal violet, fetal calf serum, and antibiotics for cell cultures and sample buffer Laemmli were from Sigma-Aldrich (Dorset, UK); Epikuron^®^200 was from Cargill (Minneapolis, MN, USA); Cremophor^®^RH60 was from BASF (Ludwigshafen am Rhein, Germany); taurocholic acid sodium salt was from ICN Biomedicals (Aurora, OH, USA); potassium phosphate monobasic, potassium phosphate dibasic, and dioctyl sodium sulfosuccinate (AOT) were from Merck (Darmstadt, Germany), agar and trilaurin were from Alfa-Aesar (Ward Hill, MA, USA); hyaluronic acid sodium salt 1600 kDa (HA) was from Farmalabor (Barletta, Italy); Avastin^®^ Roche (Basilea, Switzerland) was gently purchased from Molinette Central Hospital (Turin, Italy); sodium chloride (NaCl) and ethanol were from Carlo Erba (Val De Reuil, France). Boyden chamber filters were from Neuro Probe (BIOMAP snc, Milan, Italy) and Matrigel was from BD Biosciences. The retinal pigment epithelial ARPE-19 cell line (ATCC-CRL-2302) and the DMEM:F12 medium were purchased from ATCC^®^ (Manassas, VA, USA).

Deionized water was obtained using a MilliQ water purification system (Millipore, Bedford, MA, USA).

### 2.2. Preparation and Characterization of Lipid NPs

NPs were prepared using the “cold dilution of microemulsion” technique [13,14]. Briefly, O/W microemulsions (µEs) were prepared by solubilizing trilaurin and Epikuron^®^200 in water-saturated ethyl acetate (s-EA); then, EA-saturated water (s-water), surfactants, and co-surfactants were added, and the mixture was stirred until a transparent formulation was obtained. µE compositions are reported in Table 1.

µEs were diluted with water to obtain the precipitation of NPs, and then HA was added only in NPs 4–5 and 9–10.

NPs were purified using gel-filtration chromatography. Briefly, 0.5 mL of NPs was layered on the head of an agarose cross-linked gel (Sepharose^®^CL4B) 9 × 1.2 cm^2^ (length × diameter) column, and it was eluted with 0.3 M of NaCl to separate the BVZ-loaded NPs from the free BVZ and from other microemulsion components.

Samples employed in cell viability assays were prepared in sterile conditions by working under a vertical laminar flow hood. All components were previously sterilized in a steam autoclave or, when this was not possible, under UV rays.

### 2.3. BVZ-loaded NP Preparation

To obtain BVZ-loaded NPs, O/W µEs containing the hydrophobic AOT-BVZ ion pair [16] (µE6–µE10, Table 1) were prepared by introducing appropriate amounts of EA-saturated Avastin^®^ in s-water and then following the procedure reported above for blank NPs, adding AOT at a 1:150 BVZ:AOT molar ratio. The final step was the precipitation of NPs by µE water dilution and, when required, the solubilization of HA in aqueous suspension.

For simplicity, in the present paper, we will indicate the AOT-BVZ ion pair entrapped in NPs as BVZ.

### 2.4. NP Characterization

#### 2.4.1. Shape and Size

NP suspensions were observed by scanning electron microscopy (SEM, Carl Zeiss AG EVO50 XVP, Oberkochen, Germany) to determine the shape and size of NPs; 25 µL of 1:10-diluted NP suspensions were deposited on copper stubs, and the samples were sputter-coated with a 20 nm gold layer. In order to obtain figures with good resolution, only the biggest NPs were photographed and measured.

NP sizes and polydispersity indexes (P.I.) were also determined by the dynamic laser light scattering technique (DLS, Brookhaven Instruments, New York, NY, USA). NP suspensions were diluted with water to obtain the proper concentration for the determination. Size measurements were obtained at an angle of 90° at 25 °C using the intensity method.

#### 2.4.2. BVZ Entrapment Efficiency

BVZ entrapment efficiency (EE%) was determined both by HPLC analysis and by sodium dodecyl sulfate polyacrylamide gel electrophoresis (SDS-PAGE).

HPLC analysis—The amount of BVZ loaded into NPs was calculated based on the difference between the amount of BVZ introduced into the µE and that not entrapped in NPs. Briefly, during the purification process of BVZ-loaded NPs with gel-filtration chromatography, the fractions containing free BVZ were collected and analyzed by HPLC. The EE% was expressed as the percentage amount of BVZ loaded into NPs versus the total amount used in µE preparation. The gel-filtration chromatography apparatus consisted of an agarose cross-linked gel (Sepharose^®^CL4B) 9 × 1.2 cm (length × diameter) column as a stationary phase and 0.3 M of NaCl as a mobile phase. The separation capability of the gel-filtration chromatography column was previously verified by eluting sequentially an aliquot of free-BVZ solution and an aliquot of NP suspension. The collected fractions (each of 0.5 mL) were analyzed to assess the presence of BVZ (by HPLC) or of NPs (measuring the transmittance in a spectrophotometer).

Briefly, 0.5 mL of NPs was layered on the top of the 10 mL column and eluted by gravity-adding 0.3 M of NaCl. The free BVZ concentration in non-containing NP fractions was measured by the HPLC method described in Section 2.9.

SDS-PAGE-BVZ entrapment was confirmed using SDS-PAGE, as described in Section 2.6.1. The electrophoretic bands of BVZ-NPs before and after gel-filtration chromatography purification were analyzed with the KODAK 1D program. The ratio between the pixel of the purified NP and non-purified NP bands × 100 expresses the BVZ EE%.

To verify that BVZ was effectively loaded into NPs, gel-filtration chromatography followed by SDS-PAGE (Section 2.6.1) was performed both on BVZ-NPs and on a solution of BVZ. Briefly, 2 mg mL^−1^ of BVZ solution (the same BVZ concentration used in µE6) was prepared in the presence of taurocholic acid sodium salt, Cremophor^®^RH60, and propylene glycol (sol A). Then, 0.5 mL of sol A was loaded on the gel-filtration chromatography, eluted by the addition of 0.3 M of NaCl, and the collected fractions of 0.5 mL were analyzed. The same procedure was repeated for NP6 (µE 6, see Table 1).

### 2.5. NP Physical Stability

Sizes and Zeta potentials of different NP samples were monitored for up to 90 days after preparation, according to the previously described methods, to evaluate the overtime physical stability of the NPs.

### 2.6. BVZ Stability

#### 2.6.1. SDS-PAGE

Samples were diluted with a 2× sample buffer (0.125 M Tris-HCl pH 6.8, 4% SDS, 20% glycerol, 0.02% bromophenol blue, and 5% beta-mercaptoethanol) and were separated by SDS-PAGE 10% polyacrylamide gel. PageRuler Prestained Protein Ladder (Thermofisher,**** Waltham, MA, USA) was included on the gel. Gels were stained with InstantBlue Ultrafast Protein Stain (Sigma).

To determine the BVZ stability over time, the SDS-PAGE of the samples was repeated for up to 90 days.

#### 2.6.2. Circular Dichroism

Circular dichroism (CD) spectra were recorded from 195 to 260 nm at 20 °C on a J-810 spectropolarimeter (JASCO Europe, Cremella, Italy), equipped with a Peltier system for temperature control. Measurements were performed in a 1 cm path-length cuvette on 0.25 mg mL^−1^ of commercially available BVZ in 0.9% of NaCl, in the absence and in the presence of surfactants and of the AOT counterion at the same concentrations used in μE1.

#### 2.6.3. Cell Motility Assay

Human umbilical vein endothelial cells (HUVECs) were isolated from umbilical veins by trypsin treatments (1%) and cultured in M199 Medium with the addition of 20% of FCS, 100 UI mL^−1^ of penicillin, 100 µg mL^−1^ of streptomycin, 5 UI mL^−1^ of heparin, 12 µg mL^−1^ of bovine brain extract, and 200 mM of glutamine. The HUVECs were grown to confluence in flasks and used at the 2nd–5th passages. The use of HUVECs was approved by the Ethics Committee of the “Presidio Ospedaliero Martini” of Turin and conducted in accordance with the Declaration of Helsinki. Written informed consent was obtained from all donors.

In the Boyden chamber-migration assay, 1000 HUVEC cells were plated onto the apical side of 50 μg mL^−1^ of Matrigel-coated filters (8.2 mm diameter and 0.5 μm pore size) in a serum-free medium with or without BVZ (5–20 μM), BVZ-loaded NP9 (0.06–0.12–0.24 μM), or free NPs (NP4). A medium containing 10 ng mL^−1^ of VEGF-α was placed in the basolateral chamber as a chemoattractant. The chamber was incubated at 37 °C under 5% of CO_2_. After 18 h, cells on the apical side were wiped off with Q-tips. Cells on the bottom of the filter were stained with crystal-violet and all were counted with an inverted microscope (magnification 40×). The results are expressed as percentages of migration inhibition compared to the VEGF-α chemotactic stimulus. The control migration was 45 ± 4 cells per microscope field (*n* = 5).

### 2.7. In Vitro Release Study

The in vitro release of BVZ from NPs was determined by the nonequilibrium dialysis method [17] using a multicompartmental rotating cell system consisting of donor and receptor compartments of equal volume (1.5 mL) separated by a membrane with 0.1 µm pores.

The receiving medium was 0.125% *w*/*w* HA in water. With opportunely diluted Avastin^®^ in 0.125% *w*/*w* HA aqueous solution, NP9 and NP10 suspensions were used as donor formulations in separate experiments. At fixed times, the receptor solution was withdrawn, and the compartment was refilled with fresh receiving medium, obtaining sink conditions. The BVZ concentration in the receiving medium was determined by HPLC.

Diffusion rate constants were calculated from the slope of the straight line obtained by plotting the amount of BVZ released versus time.

### 2.8. Cell Viability Assay

The biocompatibility of NPs toward retinal pigment epithelial ARPE-19 cells was evaluated by Sulforhodamine B colorimetric viability assay (SRB assay).

ARPE-19 cells were routinely grown in the DMEM:F12 medium, with the addition of 10% (*v*/*v*) of fetal bovine serum and 1% (*v*/*v*) of penicillin-streptomycin, and were maintained in standard conditions (37 °C, 5% CO_2_, and 95% humidity).

Next, 10,000 cells were seeded into 96-well plates. After 24 h, cells were incubated for 72 h in triplicate wells, with the NP4 and NP9 properly diluted (1:50, 1:100, and 1:200) in culture medium, to produce BVZ concentrations of 20, 10, and 5 μg mL^−1^, respectively. BVZ at the same concentrations were used as a reference.

The SRB assay was carried out as previously described [18]. The values refer to the means of three separate experiments each carried out in triplicate.

### 2.9. HPLC Analysis

HPLC analysis was performed using an LC9 pump (Shimadzu, Tokyo, Japan) with a Teknokroma HPLC C8 5 μm × 25 cm × 4.6 mm column and a C-R5A integrator (Shimadzu, Tokyo, Japan); mobile phase: water/CH_3_CN/TFA 64/36/0.1 (flow rate: 1.0 mL min^−1^). The analysis was performed at 75 °C and monitored at λ_ex_ = 280 nm and λ_em_ = 360 nm. The retention time was 3.3 min.

The limit of quantification, defined as the lowest BVZ concentration in the curve that can be measured routinely with acceptable precision and accuracy, was 1.5 µg mL^−1^, and the limit of determination, defined as the lowest detection limit, was 0.5 µg mL^−1^ (signal-to-noise ratio >2.0).

### 2.10. Data Analysis

The data are shown as means ± SEM (standard error mean standard deviation/number of replicates). Statistical analyses were performed with GraphPad Prism 3.0 software (La Jolla, CA, USA) using the one-way ANOVA and Dunnett’s test. Values of *p* < 0.05 were considered statistically significant.

## 3. Results and Discussion

The current standard of care for the treatment of eye posterior segment diseases consists of intravitreal injections that allow the drug to directly reach the site of action. BVZ, a monoclonal antibody used in the treatment of age-related macular degeneration and of other segment posterior pathologies, is injected intravitreally every 30 days as off-label therapy, even if its half-life is described to be 4–5 days [8]; the frequent administrations, essential to achieve therapeutic effects, can determine some adverse effects such as retinal detachment and endophthalmitis. A drug delivery system able to release BVZ slowly for a prolonged time could be useful to reduce the administration frequency, therefore minimizing adverse effects and ameliorating the patient’s therapy acceptance.

In this paper, BVZ-loaded lipid NPs suitable for injection in the vitreous humor were prepared. As BVZ is a hydrophilic molecule, the hydrophobic ion pairing technique was adopted to obtain an ion pair between BVZ and AOT to be retained in the inner oil phase of μEs and to be incorporated within NPs.

The feasibility of lipid NP intravitreal injection was assessed by the numerous data reported in the literature; indeed, different authors indicated that, after intravitreal injections, the turbidity of NPs neither affects the vision nor determines autoimmune phenomena and/or alterations in the behavior of ophthalmic cells [19,20].

### 3.1. NP Characterization

BVZ-loaded NPs were prepared using a “cold dilution of microemulsion” technique. Starting from a previous formulation study regarding the optimization of μE composition [15], selecting the most suitable components, different empty and BVZ-loaded systems were prepared. Dioctyl sodium sulfosuccinate (AOT) was introduced in the formulations to form a hydrophobic ion pair with BVZ, which was easily dissolved in the oil phase of the μE and, consequently, in the lipid matrix of NPs; moreover, different types and amounts of surfactants/co-surfactants were screened.

With the aim of obtaining formulations carrying BVZ in a concentration high enough to test its activity in vitro, different amounts of BVZ and different NP suspension dilutions were used.

#### 3.1.1. Shape and Size

**Figure 1:**
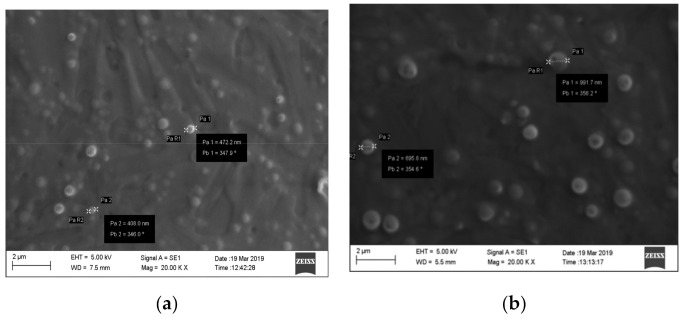
(**a**,**b**) SEM images of NP3 and NP8 at 20,000× magnification.

Blank NPs or BVZ-loaded NP suspensions were characterized by SEM observation. In Figure 1a,b, SEM images of blank NP3 and NP8 (containing the highest BVZ concentration used (Table 1) are shown.

From the images, it can be remarked that both NPs had a spherical shape and were homogeneously dispersed without aggregates or crystals. SEM analysis indicated that the loading of BVZ (NP8) increased the mean diameter of NPs, as also reported in the literature [21]; a possible explanation can be related to the high molecular dimension of BVZ that, after entrapment in the μE disperse phase, determines a size increase in the resulting NPs.

Size analysis was also performed by DLS. In Table 2, the mean sizes, P.I.s, and Zeta potentials of NP1–NP10 are reported.

As shown in Table 2, all NPs had mean diameters in the 280–430 nm range and P.I.s between 0.2 and 0.3. The DLS results confirmed, although in a less evident way than SEM analysis, the higher mean sizes of all BVZ-loaded NPs compared to blank NPs.

The Zeta potential of all NPs was clearly negative with values between −31 and −17 mV; the negative charge could be due to the presence of soya lecithin (Epikuron^®^200) that precipitates with trilaurin following μE dilution, both constituting the NP matrix. Moreover, as can be seen, the Zeta potential values of NP3 and NP8 were significantly less negative than all other formulations, probably due to the absence of taurocholic acid sodium salt, which contributed to the negative superficial charge of other formulations, while the Zeta potentials of NP9 and NP10 were much more negative due to the presence of HA. Thus, it can be assumed that the presence of HA could increase the overtime physical stability of NPs. The entrapment of BVZ into NPs does not influence Zeta potential values in a significant way, suggesting that it was completely incorporated into the solid matrix.

Moreover, it is well-known from the literature data [22,23] that the negative surface charge overcomes the vitreous binding characteristic of cationic NPs.

#### 3.1.2. BVZ Entrapment Efficiency

**Figure 2:**
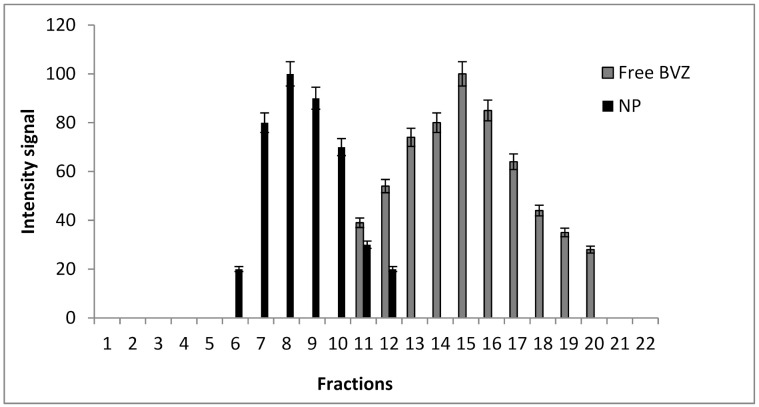
Gel-filtration chromatograms for NPs (black) and free BVZ (grey).

As BVZ can aggregate and/or degrade in the presence of high amounts of organic solvent, the use of organic solvents, which are the only ones able to solubilize lipid NPs, must be avoided. Therefore, BVZ EE% determination was carried out by analyzing BVZ in the continuous phase of the suspension using gel-filtration chromatography to separate BVZ-loaded NPs from free BVZ. Gel-filtration chromatography column calibration was carried out by loading separately BVZ solution and NP suspension to identify the fractions containing free BVZ and assessing the absence of any significant overlapping. In Figure 2, the elution fractions of NPs (black) and of free BVZ (grey) are reported.

A satisfactory separation between NPs and free-BVZ was obtained with a slight overlap between fraction 11 and 12. Fractions from 12 to 20 were considered as free BVZ fractions after centrifuging them to remove the residual NPs before BVZ determination.

**Figure 3:**
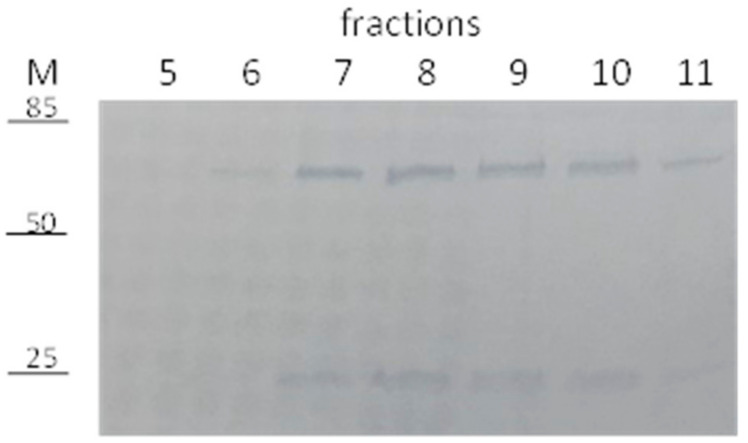
SDS-PAGE of 5–11 fractions NP6.

BVZ-NP6 were loaded on the gel-filtration column and 500 μL fractions were collected, as described above, to separate NPs and free-BVZ. The fractions containing NPs were analyzed by SDS-PAGE to assess the presence of BVZ (Figure 3).

As shown, the BVZ presence is evident in the fractions from 6 to 11 with the major amount in the NP elution fractions 7–10, demonstrating the co-presence of BVZ in the BVZ-NP6-purified fractions. As a control, sol A (BVZ in the presence of all surfactants and co-surfactants) was loaded on the gel-filtration chromatography, and 500 μL fractions were collected and analyzed by SDS-PAGE. BVZ was detected only in the 11–20 fractions (data not reported). These data all together strongly suggest the successful entrapment of BVZ in NPs.

The BVZ EE% was determined both by SDS-PAGE and HPLC analysis by comparing the concentration of BVZ in non-purified NPs with that in gel-filtration chromatography-purified NPs. In Table 3, the EE% of all NPs (from 6 to 10), determined by both methods, are reported.

From these results, it is clear that the ability of NPs, obtained with the method described in this paper, to entrap BVZ as a hydrophobic ion pair is more than satisfying as BVZ EE% ranges from 50% in NP6 to 93% in NP10, as confirmed by the data obtained by both analytical methods (HPLC and SDS-PAGE).

The BVZ EE% seems to be influenced by µE composition in a significant way. In particular, the presence of high amounts of taurocholic acid sodium salt in µE6 would lead to a considerable decrease in the amount of entrapped BVZ, probably due to the formation of micelles in the aqueous phase and to their solubilizing capacity; indeed, in NP6, the BVZ EE% was 50.5%, compared with values higher than 90% for NP7 and NP8, which were formulated with small amounts or without bile salt. On the contrary, the addition of HA after NP precipitation did not influence BVZ entrapment: BVZ EE% in NP9 and NP10 calculated by both methods was always >95%.

Because of their high entrapment efficiency, NP8, NP9, and NP10 were selected as formulations to be tested in the following studies.

### 3.2. NP Physical Stability

Mean sizes and Zeta potentials of NP8, NP9, and NP10 were monitored for up to 3 months to verify the stability of NPs over time. In Table 4, the mean diameters and Zeta potentials of the samples stored at different temperatures are reported.

As shown, all NPs were stable over a three-month period; only small increases in size were registered during the storage at 36 °C, simulating the ocular temperature. HA introduced in NP9 and NP10 seemed to improve the overtime stability of the NP size; following the addition of HA, the viscosity of NP dispersions was increased and a HA microgel with a 3D network-like structure was formed, inducing more stable dispersions, as also reported by Chen et al. [24].

The Zeta potential, too, maintained a clearly negative value throughout the period considered.

In this study, the released drug was not quantified. This determination will be carried out in the near future. Moreover, a freeze-drying method is being developed to preserve the samples over time.

### 3.3. BVZ Stability

#### 3.3.1. SDS-PAGE

NP-entrapped and free BVZ stabilities were investigated by SDS-PAGE analysis at different conditions and at different time points.

**Figure 4:**
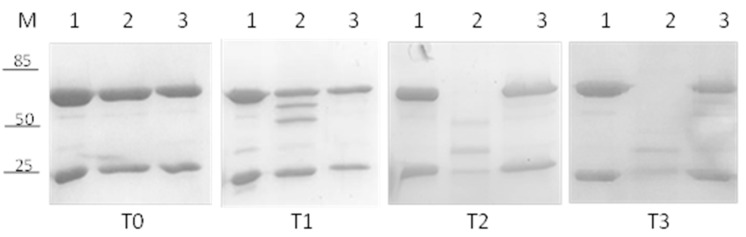
SDS-PAGE analysis to assess the stability of commercially available BVZ (Avastin^®^) at different time points: (M) Marker, 1 BVZ at 4 °C, 2 BVZ in phosphate buffer, 3 BVZ in vitreous humor-like environment. T0 (freshly prepared), T1 (14 days), T2 (30 days), and T3 (60 days).

As a control, the stability of commercially available BVZ (Avastin^®^) was tested. In Figure 4, the results at 36 °C of free BVZ diluted in phosphate buffer (lane 2) and in a vitreous humor-like environment (lane 3) at different time points are reported: 0 (panel T0), 14 (panel T1), 30 (panel T2), and 60 (panel T3) days. As a negative control, commercial BVZ stored at 4 °C was used (lane 1).

BVZ in the vitreous humor-like environment was stable for up to 60 days, considering that no degradations of heavy and/or light chains were visible (Figure 4, Lane 3 T0, 1, 2 and 3), whereas, surprisingly, a significant degradation of heavy chains was observed when BVZ was diluted in phosphate buffer (already after 14 days, Lane 2 T1), followed quickly by the light chain too (lane 2 T2). After 30 days, neither heavy nor light chains were visible (30 and 60 days, Lane 2 T2 and 3). BVZ at 4 °C, used as a control, showed no visible degradation even after 60 days (Lane 1 T3).

Subsequently, the stability of BVZ was analyzed when entrapped in NPs. A considerable degradation of both heavy and light chains was observed at 30 days when BVZ was loaded into NP8 and stored at 36 °C (data not shown).

**Figure 5:**
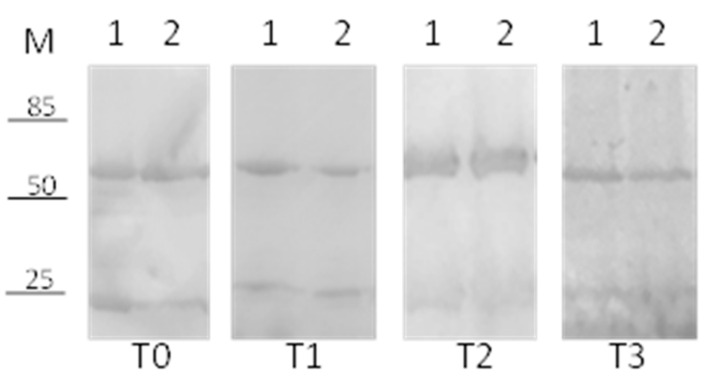
SDS-PAGE analysis to assess the effect of hyaluronic acid. (M) Marker, 1 NP10 (0.25% HA) stored at 36 °C, 2 NP9 (0.125% HA) stored at 36 °C. T0 (freshly prepared); (T1 (40 days); T2 (60 days); T3 (90 days).

Considering its positive effect on the free-BVZ stability, HA, an FDA-approved polysaccharide, widely used as an excipient for ophthalmological dosage forms [25], was added to the BVZ-NP suspension at two different concentrations (0.125% and 0.25% Figure 5, Lanes 1 and 2, respectively) to evaluate its effect on BVZ stability when entrapped in NPs at 36 °C.

In Figure 5, a significant increase in BVZ stability was detectable in NPs (Lane 1 and 2, T0, 1, 2, and 3) until 90 days, probably due to the presence of HA. No difference in stability was noted with the variation in the HA concentration.

#### 3.3.2. Circular Dichroism

As the use of surfactants and co-surfactants in the µE composition might promote protein unfolding, the BVZ stability was checked in their presence. The possible formation of oligomers was excluded by gel filtration associated with SDS-PAGE analysis. In fact, SDS-PAGE of the BVZ solution in the presence of all surfactant/co-surfactants showed BVZ bands only in the fractions 11–20, as well as commercial Avastin^®^ (data not reported). The absence of BVZ signals in previous gel-filtration chromatography fractions indicates that no BVZ aggregates formed.

As BVZ loading in NPs is proposed as a tool for prolonged release treatment, it is mandatory to test the ability of BVZ to retain its three-dimensional structure when exposed to surfactants and co-surfactants in the µE composition.

Secondary structural analysis by CD was carried out to investigate the secondary structure stability of BVZ when associated with NPs. CD spectroscopy directly on the BVZ-NP suspension was impaired, as significant background noise was detected. To address this problem, the secondary structural analysis of BVZ was carried out in the presence of several NP components that could affect its conformation.

**Figure 6:**
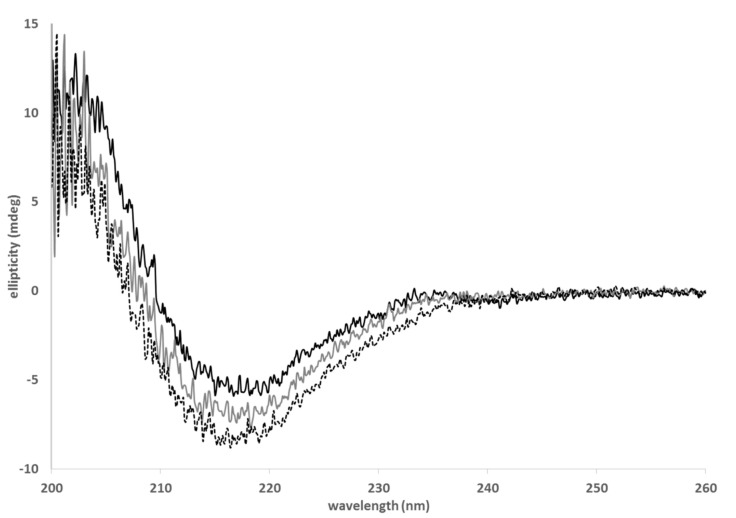
CD spectra of commercial BVZ (black), of BVZ in the presence of surfactants (grey), and of BVZ in the presence of surfactants and AOT (dotted black).

In Figure 6, the CD spectra of BVZ in the presence of surfactants (grey curve) and of AOT counterions (dotted black curve) are shown. Commercially available BVZ (Avastin^®^ 25 mg mL^−1^ 1:100 diluted) was recorded as a control (black line). The BVZ spectrum presented a minimum between 210 and 220 nm.

This curve shape is associated with proteins containing mostly β-sheet secondary structure elements, as for immunoglobulins (such as BVZ). Indeed, this large superfamily of proteins is composed of 70% of antiparallel β-sheets [26].

BVZ with surfactants (BVZ-surf-grey curve) and BVZ in the presence of AOT (BVZ-surf-AOT-light-grey curve) present a similar trend to the curve generated by Avastin^®^ (black curve). This strongly indicates that BVZ retains its structural integrity even in the presence of surfactants and counterions used for the CD experiments. These results greatly support that BVZ is correctly folded when entrapped in NPs.

### 3.4. In Vitro Release Study

**Figure 7:**
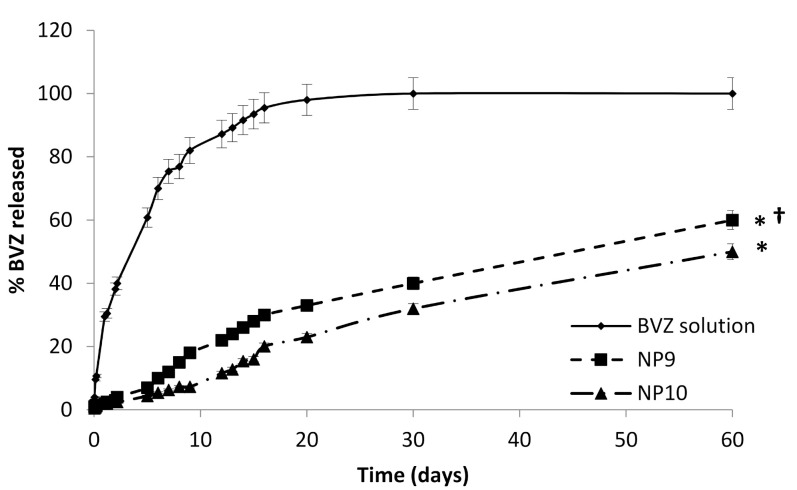
BVZ release profiles. Asterisks * refer to statistically significant differences of BVZ released from NP9 and NP10 vs. BVZ solution (* *p* < 0.05). The cross (†) refers to statistically significant differences of BVZ released from NP9 vs. NP10 († *p* < 0.05).

The study of BVZ release was conducted on NP9 and NP10 using as a reference a diluted commercial BVZ solution in the presence of 0.125% *w*/*w* HA (Figure 7).

No burst effect was observed from both NP9 and NP10, suggesting an actual incorporation of BVZ in the lipid matrix; in fact, a burst effect release pattern usually occurs in nanostructured systems with high amounts of unentrapped or surface-attached drugs.

The apparent diffusion constants calculated according to Garret and Chemburkar [27] were obtained from the slope of the straight section of the curve obtained by plotting the BVZ diffused amount vs. evaluated time curve permeation profiles. The apparent diffusion constants were 6.80 × 10^−4^ cmh^−1^ for NP9, 4.56 × 10^−4^ cm/h for NP10, and 1.10 × 10^−2^ cm h^−1^ for the diluted commercial BVZ solution, respectively.

The release data of BVZ solution and of BVZ-loaded NPs (NP9 and NP10) were quite different; the diffusion of BVZ from the solution was very fast in the initial days and reached 100% in 20 days according to first-order kinetics.

On the contrary, BVZ diffused more slowly from NP9 and NP10, showing pseudo-zero-order kinetics maintained throughout the duration of the diffusion study (60 days). According to these results, the diffusion of BVZ from the lipid matrix is the main rate-determining step for the release, and the different apparent diffusion constants from solution and NPs can, thus, be attributed to the entrapment of BVZ in the lipid core.

Therefore, the in vitro release results both confirmed BVZ entrapment in NPs and the feasibility of their future exploitation as intravitreal slow-release systems.

The slight difference in the BVZ apparent diffusion coefficient from NP9 and NP10 (*p* ˂ 0.05) could be attributed to the different amounts of HA that modify the sample viscosity. These results demonstrate that a slow and prolonged release of BVZ from NPs is possible. Due to the BVZ instability over time in NP8, not containing HA, the release study from this sample was not realized.

### 3.5. Cell Assays

The biocompatibility of blank and BVZ-loaded NPs (NP4 and BVZ-NP9, respectively) was tested on retinal pigmented epithelium ARPE-19 cells. This cell line was employed in this study as a model of ocular epithelial cells. Free BVZ was used as a reference.

**Figure 8:**
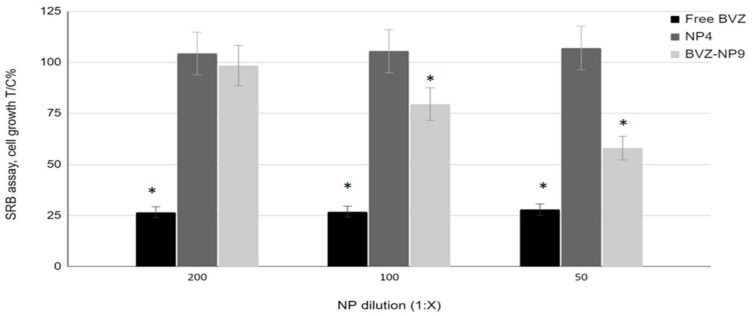
Effect of BVZ, NP4 (blank NP), and NP9 (BVZ-NP) on ARPE-19 cell proliferation after 72 h of incubation. On the *X*-axis, the NP dilutions 1:200, 1:100, and 1:50 correspond to 5, 10, and 20 µg/mL BVZ concentrations. Blank NP4 was diluted as NP9. Free BVZ was diluted appropriately to obtain the same BVZ concentration. The cell growth is expressed as % T/C (mean OD of treated cells/mean OD of control cells × 100). Values refer to the means ± SD (*n* = 3 wells/condition) of three independent experiments. Asterisks * indicate statistically significant differences of BVZ and NP9 vs. NP4-treated cells at the same concentrations (* *p* < 0.05).

The cells were incubated for 72 h with each sample at different BVZ concentrations (from 5 to 20 μg mL^−1^) and the cell growth was evaluated by SRB assay (Figure 8).

As shown in Figure 8, the viability of ARPE-19 cells after 72 h of incubation was not affected by the blank NP4, even at the highest concentration tested. This finding suggests a good biocompatibility of the basic ingredients of the NP system.

Instead, a significant decrease in cell viability was observed for free BVZ at all concentrations. The effect of BVZ loaded into NPs was observed only at the highest concentration tested. At 5 μg mL^−1^, it did not influence cell growth, but at 10 and 20 μg mL^−1^, it caused a 10 and 40% loss in viability, respectively. To demonstrate that the lower effect of NP-loaded BVZ on ARPE 19 is not due to a loss of BVZ activity after the entrapment, a simple cell motility assay was performed. In this study, the motility of HUVEC cells after incubation with free BVZ, NP4, or BVZ-NP9 in the presence of a VEGF-α chemotactic stimulus was evaluated. Free BVZ tested at a 1.5 mg mL^−1^ concentration inhibited migration by about 60%, blank NP4 had no effect on cell migration, while BVZ-NP9, tested at about a 50 times lower BVZ concentration (30 µg mL^−1^), induced a migration inhibition of about 40% (data not shown). Indeed, BVZ loaded into NPs maintains its activity and BVZ-NP9 was even at least 50 times more active than the free substance.

As was demonstrated, as entrapped BVZ maintained its activity, the minor effect of loaded BVZ compared to free BVZ on ARPE-19 could be explained by its slow release from NPs, as shown in the release studies previously reported.

## 4. Conclusions

The main purpose of this article was to develop an innovative and biocompatible de-livery system of BVZ, based on lipid nanoparticles, suitable for intravitreal injection, as a precondition to undertake further studies aiming to improve its intraocular bioavailability, avoiding patient discomfort due to repeated intravitreal administrations.

The use of the formulation technology called “cold dilution of microemulsions” allowed a new carrier to be obtained for BVZ administration, able to entrap a great number of antibodies and to release them slowly in vitro. The entrapment of antibodies in lipid systems is generally a critical point due to their aqueous solubility, as well as their ease of degradation and loss of structure during the preparation process. In this work, the use of AOT as a counterion of BVZ to exploit the hydrophobic ion pairing resulted in the winning strategy to load a great number of antibodies into NPs. Moreover, the observation of the increased BVZ stability in a vitreous humor-like environment was crucial to realize that the introduction of HA in the NP suspension could improve the antibody stability. Thus, in this work, a stable BVZ-loaded system was obtained. In addition, several studies have shown that BVZ was released slowly from the lipid matrix and that this system was biocompatible.

Soon, this system will be injected into the ocular flow cell [18] to study the in vitro BVZ clearance and distribution in the eye.

In conclusion, the obtained results are significant and open new possibilities of effective and minimally invasive treatments of intraocular diseases.

## Figures and Tables

**Table 1:** Composition of blank (µE1–µE5) and BVZ-loaded (µE6–µE10) microemulsions (µEs); µE6–µE7 containing 2 mg of BVZ (corresponding to 80 µL of ethyl acetate (EA)-saturated Avastin^®^); µE8–µE10 containing 4 mg of BVZ (corresponding to 160 µL of EA-saturated Avastin^®^). * The amount of s-water was 620 μL in µE6–µE7 and 540 μL in µE8–µE10 as the volume of Avastin^®^ solution was subtracted.

	Ingredients (mg)	µE1 and 6	µE2 and 7	µE3 and 8	µE4 and 9	µE5 and 10
	s-EA	202	202	202	202	202
**MICROEMULSION**	Trilaurin	60	60	60	60	60
	Benzyl alcohol	75	-	-	-	-
	AOT	0.9	0.9	1.8	1.8	1.8
	Epikuron^®^200	150	150	150	150	150
	Taurocholic acid sodium salt	50	10	-	-	-
	Cremophor^®^RH60	65	45	45	45	45
	Propylene glycol	-	-	200	200	200
	s-water *	700 μL	700 μL	700 μL	700 μL	700 µL
	Dilution water	5 mL	5 mL	3 mL	3 mL	3 mL
	HA after dilution	-	-	-	5	10

**Table 2:** Mean diameters, polydispersity indexes, and Zeta potentials of blank nanoparticles (NPs) (NP1–NP5) and BVZ-loaded NPs (NP6 *–NP10 *). Asterisks * refer to formulations with BVZ.

Formulation	Mean Diameter (nm)	P.I.	Zeta Potential (mV)
NP1	398.6 ± 2.4	0.298	−23.54 ± 1.36
NP2	326.9 ± 2.9	0.237	−21.84 ± 1.44
NP3	286.5 ± 1.2	0.281	−18.57 ± 1.08
NP4	287.4 ± 4.6	0.215	−30.87 ± 2.65
NP5	280.6 ± 4.1	0.268	−31.78 ± 3.01
NP6 *	432.1 ± 3.8	0.190	−24.20 ± 1.85
NP7 *	368.4 ± 3.7	0.281	−22.59 ± 2.15
NP8 *	385.4 ± 2.1	0.318	−17.39 ± 1.51
NP9 *	370.7 ± 4.8	0.235	−30.15 ± 3.85
NP10 *	350.5 ± 6.5	0.237	−31.58 ± 1.57

**Table 3:** BVZ entrapment efficiency.

Formulation	BVZ EE% HPLC	BVZ EE% SDS-PAGE	BVZ *w*/*w*% (mg BVZ/mg NP)
NP6	50.5 ± 1.5	53.4 ± 1.3	0.48
NP7	91.4 ± 2.7	93.5 ± 2.5	0.88
NP8	90.4 ± 2.1	92.6 ± 1.8	1.74
NP9	95.5 ± 3.2	94.2 ± 3.1	1.81
NP10	95.2 ± 2.3	93.1 ± 3.4	1.79

**Table 4:** Sizes and Zeta potentials of NP8, NP9, and NP10 for up to 90 days.

Time after Preparation	NP8 Mean Diameter (nm) Zeta Potential (mV)		NP9 Mean Diameter (nm) Zeta Potential (mV)		NP10 Mean Diameter (nm) Zeta Potential (mV)	
T0	385.4 ± 2.1 −17.39 ± 1.51		370.7 ± 4.8 −30.15 ± 3.85		350.5 ± 6.5 −31.58 ± 1.57	
Temperature	5 °C	36 °C	5 °C	36 °C	5 °C	36 °C
7 days	388.5 ± 3.2 −18.86 ± 1.58	391.2 ± 14.2 −23.60 ± 2.59	365.3 ± 4.4 −30.15 ± 2.77	364.9 ± 6.2 −30.20 ± 2.91	347.3 ± 11.6 −31.30 ± 2.37	350.9 ± 4.2 −31.66 ± 3.11
14 days	405.1 ± 14.9 −18.71 ± 1.86	403.5 ± 13.4 −18.73 ± 2.18	362.9 ± 1.3 −29.88 ± 2.40	370.1 ± 6.3 −29.54 ± 3.87	353.9 ± 1.3 −30.94 ± 2.10	358.1 ± 11.3 −30.54 ± 3.25
21 days	407.9 ± 4.7 −18.73 ± 1.01	425.8 ± 7.9 −18.93 ± 1.95	371.9 ± 8.8 −29.15 ± 2.75	374.9 ± 6.1 −28.83 ± 3.28	361.9 ± 13.8 −30.03 ± 1.40	384.9 ± 8.1 −30.23 ± 1.24
30 days	429.4 ± 31.5 −17.8 ± 2.13	448.8 ± 20.5 −18.43 ± 1.13	378.8 ± 8.7 −28.95 ± 2.32	386.8 ± 7.5 −28.25 ± 2.60	368.8 ± 8.7 −29.86 ± 2.09	406.8 ± 10.5 −29.69 ± 2.63
60 days	471.9 ± 12.2 −17.19 ± 2.04	497.6 ± 26.8 −16.58 ± 1.52	392.8 ± 7.6 −28.22 ± 2.34	400.5 ± 15.1 −28.11 ± 2.65	380.8 ± 10.6 −29.38 ± 1.32	420.5 ± 25.4 −29.01 ± 2.10
90 days	497.5 ± 29.5 −17.31 ± 1.89	532.7 ± 23.8 −15.45 ± 1.85	415.8 ± 11.4 −28.01 ± 3.12	420.5 ± 21.3 −27.98 ± 1.95	402.7 ± 12.2 −28.37 ± 2.29	433.6 ± 43.5 −28.76 ± 0.81
